# Bereavement by suicide as a risk factor for suicide attempt: a cross-sectional national UK-wide study of 3432 young bereaved adults

**DOI:** 10.1136/bmjopen-2015-009948

**Published:** 2016-01-25

**Authors:** Alexandra L Pitman, David P J Osborn, Khadija Rantell, Michael B King

**Affiliations:** 1 Division of Psychiatry, University College London, London, UK; 2 Camden and Islington NHS Foundation Trust, St Pancras Hospital, London, UK; 3 Institute of Neurology, University College London, London, UK

**Keywords:** bereavement, EPIDEMIOLOGY, suicide attempt, unnatural causes, stigma

## Abstract

**Objectives:**

US and UK suicide prevention strategies suggest that bereavement by the suicide of a relative or friend is a risk factor for suicide. However, evidence is lacking that the risk exceeds that of any sudden bereavement, is specific to suicide, or applies to peer suicide. We conducted the first controlled UK-wide study to test the hypothesis that young adults bereaved by suicide have an increased risk of suicidal ideation and suicide attempt compared with young adults bereaved by other sudden deaths.

**Design:**

National cross-sectional study.

**Setting:**

Staff and students at 37 UK higher educational institutions in 2010.

**Participants:**

3432 eligible respondents aged 18–40 exposed to sudden bereavement of a friend or relative after the age of 10.

**Exposures:**

Bereavement by suicide (n=614), by sudden unnatural causes (n=712) and by sudden natural causes (n=2106).

**Primary outcome measures:**

Incident suicidal ideation and suicide attempt.

**Findings:**

Adults bereaved by suicide had a higher probability of attempting suicide (adjusted OR (AOR)=1.65; 95% CI 1.12 to 2.42; p=0.012) than those bereaved by sudden natural causes. There was no such increased risk in adults bereaved by sudden unnatural causes. There were no group differences in probability of suicidal ideation. The effect of suicide bereavement was similar whether bereaved participants were blood-related to the deceased or not. The significant association between bereavement by suicide and suicide attempt became non-significant when adding perceived stigma (AOR=1.11; 95% CI 0.74 to 1.67; p=0.610). When compared with adults bereaved by sudden unnatural causes, those bereaved by suicide did not show significant differences in suicide attempt (AOR=1.48; 95% CI 0.94 to 2.33; p=0.089).

**Conclusions:**

Bereavement by suicide is a specific risk factor for suicide attempt among young bereaved adults, whether related to the deceased or not. Suicide risk assessment of young adults should involve screening for a history of suicide in blood relatives, non-blood relatives and friends.

**Strengths and limitations of this study**

- We conducted a large population-based national survey of young adults, using a precise sampling frame.
- We included exposures to the sudden death of any close contact, to describe the impact of suicide bereavement whether related to the deceased or not.
- Our primary outcomes were validated measures of self-reported suicidal ideation and suicide attempt occurring after the bereavement, adjusted for prebereavement suicidal behaviour and psychopathology.
- We compared bereavement by suicide with bereavement due to sudden natural causes, then separately compared those bereaved by suicide with those bereaved due to sudden unnatural death to measure the specific impact of suicide bereavement.
- Given the possibility of selection bias (favouring higher social classes) and male non-response bias, the results of this study may be more generalisable to young bereaved women than men, and to the more highly educated.

## Introduction

Suicide bereavement describes the period of grief, mourning and adjustment after a suicide death, that is experienced by family members, friends and any other contacts of the deceased affected by the loss.^1^ It is estimated to affect up to 9% of adolescents^2^ and 7% of adults^3^ annually. Since 1989, the WHO has suggested that relatives and close friends of people who die by suicide are a high-risk group for suicide.^4^ Explanations include the particular psychological trauma of a suicide loss, which involves grief and agonising self-questioning; shared familial and environmental risk; suicide contagion through the process of social modelling^1^; and the burden of stigma associated with violent losses.^1^ ^5^ Quantitative studies confirm that people bereaved by suicide and other violent deaths perceive greater stigma than other bereaved groups.^1^ Their qualitative accounts are of others’ distaste or embarrassment over the disturbing nature of an unnatural loss,^5^ and a loss of community support,^6^ ^7^ with the effect of reducing help seeking and perceptions of the support available.^7^ As stigma may be more modifiable than other potential explanatory factors, there is interest in understanding its relationship to suicide-related outcomes after negative life events.^1^

International suicide prevention strategies have placed great emphasis on the provision of support for people bereaved by suicide,^1^ despite the lack of studies confirming that risk of adverse outcomes applies beyond the effect of any sudden loss.^1^ ^8^ There is also little evidence for effective interventions after suicide.^9^ Studies comparing people bereaved by suicide with non-bereaved controls support an increased probability of suicide following any bereavement.^10^ Those using controls bereaved by non-suicide causes go further in supporting an association between sudden bereavement and suicide-related outcomes.^10^ ^11^ However, risk of hospital-treated suicide attempt is similar in adults bereaved by suicide and those bereaved by accidental deaths,^12^ suggesting that the wider risk factor is bereavement by any unnatural causes. Only study designs separating out control groups bereaved by sudden natural and sudden unnatural causes, adjusted for prebereavement psychopathology, can determine whether adverse outcomes are attributable to violent deaths or more specifically to suicide.^1^ Our recent systematic review highlighted the lack of such studies.^1^ It also found that no British studies had investigated suicide-related outcomes after suicide bereavement,^13^ and no studies using bereaved controls had measured the impact of peer suicide.^1^ This is despite widespread concern about the susceptibility of young people to social modelling of self-harm^14^ ^15^ and recent increases in suicides among young men.^16^

Our objective was to design a study that could investigate whether there is a specific association between suicide bereavement and suicide attempt by making distinct comparisons between bereavement by suicide, unnatural causes and sudden natural causes. Use of routine clinical data was precluded because these record exposure only to mortality of first-degree relatives and cohabitees, and hospital presentations of self-harm. Conversely, survey methods permit ascertainment of exposure to all bereavements, and self-reported suicidality and self-harm, and are therefore a vital tool for investigating risk of suicidal events following suicide bereavement. We therefore undertook a population-based cross-sectional survey comparing the impact of specific modes of self-reported sudden bereavement on non-fatal suicide-related outcomes.

Our primary hypothesis was that young adults in the UK who had been bereaved by suicide were at higher risk of suicidal thoughts and suicide attempts than those bereaved by other causes of sudden death. Collecting data on two control groups allowed us to address two research questions. First, comparison with adults bereaved by sudden natural causes of death took into account the sudden nature of the loss. Second, comparison with adults bereaved by sudden unnatural causes took into account the violent nature of the loss. Hypothesis 2 was that suicide bereavement would be a risk factor for four secondary clinical and occupational measures (postbereavement non-suicidal self-harm, depression, occupational drop-out and social dysfunction), reflecting policy concerns about the contribution of bereavement to workplace mental ill health and sickness absence.^17^ Hypothesis 3 was that the impact of suicide bereavement would extend beyond genetic relatedness to peer suicides, and would therefore not be modified by relatedness to the deceased. Hypothesis 4 was that any associations with clinical or occupational outcomes would be attenuated by perceived stigma, as a marker for reduced help seeking.

## Methods

### Study design and participants

We invited all young adults working or studying at UK higher education institutions (HEIs) to participate in a closed, online study about sudden bereavement: the UCL Bereavement Study. We anticipated that using the email systems of large institutions would be the best means of accessing hard-to-reach groups, particularly those not normally accessing health services, and avoiding the biases associated with recruiting a help-seeking sample.^18^ Sampling from a diverse range of colleges, universities, art and drama schools, and agricultural colleges offered unique access to a large defined sample of young adults.

All 164 HEIs in the UK in 2010 were invited to participate, following up non-responding HEIs to encourage broad socioeconomic and geographic representation. Over 20% of HEIs (37/164) agreed to take part, with a higher response (40%) from those classified as the more prestigious Russell Group of universities. This provided a sampling frame of 659 572 staff and students. All participants were invited to take part in a survey of ‘the impact of sudden bereavement on young adults’, with the aim of masking them to the specific study hypotheses. There was no accurate way of measuring response, as the denominator of bereaved people was not ascertainable using routine data or survey methods. The majority of participating HEIs agreed to send an individual email invitation with embedded survey link to each staff and student member. For reasons of sensitivity (recent staff/student deaths), 10 HEIs modified this strategy, for example, by emailing students only, using their weekly news digest email, or advertising via staff and student intranet.

Inclusion criteria were as follows: people aged 18–40 who, since the age of 10, had experienced sudden bereavement of a close friend or relative. The 18–40 age range was defined to reflect an under-researched group of great policy interest.^16^ Early childhood bereavements were excluded to minimise recall bias and restrict our focus to adult cognitive processing of life events, using the age threshold for criminal responsibility in England and Wales. A close contact was defined as ‘a relative or friend who mattered to you, and from whom you were able to obtain support, either emotional or practical’. Sudden bereavement was operationalised as ‘a death that could not have been predicted at that time and which occurred suddenly or within a matter of days’. Exposure status was classified by responses to the question: ‘Since you were aged 10 have you experienced a sudden bereavement of someone close to you due to any of the following: (1) sudden natural death (eg, cardiac arrest, epileptic seizure, stroke); (2) sudden unnatural death (eg, road crash, murder or manslaughter, work accident); (3) suicide?’ Mode of death was defined subjectively by the respondent, and not by coroner's verdict or death certificate, as perception of bereavement type was the exposure of interest.

In the case of more than one exposure, we adopted a hierarchical approach favouring those bereaved by suicide, for whom we anticipated the lowest base rate. This group were classified as suicide bereaved regardless of other exposures. Those bereaved by more than one non-suicide sudden death were asked to relate their responses to whichever person they had felt closest to, with exposure status classified accordingly.

We estimated that a minimum of 466 participants would be required in any one group (two-tailed analysis; 90% power) to detect a doubling of the UK community prevalence of lifetime suicide attempt (6.5%) in young adult samples.^19^ We chose a relatively large effect size to reflect our comparison to a non-bereaved baseline, lacking prevalence figures for bereaved UK samples.

### Procedures

The questionnaire (see online supplementary material) was designed in consultation with a group of young bereaved adults and bereavement counsellors, who identified important domains to cover in relation to the impact of bereavement, and was piloted with individuals accessing support from national bereavement support organisations. It elicited quantitative data on sociodemographic and clinical characteristics, including a personality disorder screen,^20^ and nine putative confounding variables identified a priori from existing literature and clinical judgement: age, gender, socioeconomic status, other family history of suicide (excluding index bereavement), years since bereavement, kinship to the deceased, prebereavement depression, prebereavement suicide attempt and prebereavement non-suicidal self-harm. These reflected the observed vulnerabilities of people bereaved by suicide, even before the loss,^1^ which are likely to reflect shared familial and environmental risk. We measured perceived stigma using the stigmatisation subscale of the Grief Experience Questionnaire (GEQ),^6^ with items such as ‘Since the death how often did you feel avoided by friends?’ Responses on a Likert-style scale generated scores of 5–25.

Our two main outcomes were self-reported suicidal ideation (‘Have you ever thought of taking your life, even though you would not actually do it?’)^21^ and self-reported suicide attempt (‘Have you ever made an attempt to take your life, by taking an overdose of tablets or in some other way?’).^22^ These standardised measures were taken from the Adult Psychiatric Morbidity Survey (APMS),^19^ a national seven-yearly population survey in England, and were qualified by whether these occurred before or after the sudden bereavement, to derive an incident measure.

Our four secondary measures were: postbereavement non-suicidal self-harm (self-poisoning and self-injury without suicidal intent) using a standardised APMS measure^22^ (adapted as above); depression using the Composite International Diagnostic Interview (CIDI) screen for lifetime depression^23^ (adapted as above); occupational drop-out (from work or education) using a binary measure developed for this study; and poor social functioning using the Social Functioning Questionnaire (SFQ).^24^

### Statistical analysis

We summarised sample characteristics by exposure group, using χ^2^ tests (categorical variables) and one-way analysis of variance (continuous variables). We then used multivariable regression to estimate the strength of associations between suicide bereavement and outcomes. We fitted binary models using *xtlogit* commands in Stata,^25^ with HEI as random effect, to take into account the clustering effect at HEI level. Each multivariable model included nine prespecified confounding variables, described above. Models used complete case analysis, with a significance threshold of p=0.05 for primary outcomes and p=0.01 for secondary outcomes. Our primary comparison used bereavement by sudden natural causes as the reference category, quantifying risk of adverse outcomes in adults bereaved by suicide, and in adults bereaved by sudden unnatural causes. We conducted a second comparison between adults bereaved by suicide and the reference category of adults bereaved by sudden unnatural causes.

We tested hypothesis 3, whether the effect of suicide bereavement was modified by kinship (blood-related vs non-blood-related), by adding an interaction term to all models. Hypothesis 4, whether stigma attenuated associations, was tested by including stigma scores in multivariable models.

A series of a priori defined sensitivity analyses were conducted. These assessed the robustness of our main findings when using best-case and worst-case scenarios to impute missing values, and when applying more stringent inclusion criteria (excluding participants from the 10 HEIs that had modified the stipulated recruitment method; excluding participants from the 18 HEIs with participant numbers below the median cluster size).

Finally, we conducted a set of four post hoc analyses to describe probability estimates in those bereaved by the death of an older person; in a student-only sample; and in women (as the study was underpowered to add gender as an interaction term to models); and to ascertain whether exposure to more than one mode of sudden bereavement might attenuate associations (by adding this variable to final models).

All analyses were conducted using Stata V.12 (StataCorp, Texas, USA).

## Results

**Figure 1:**
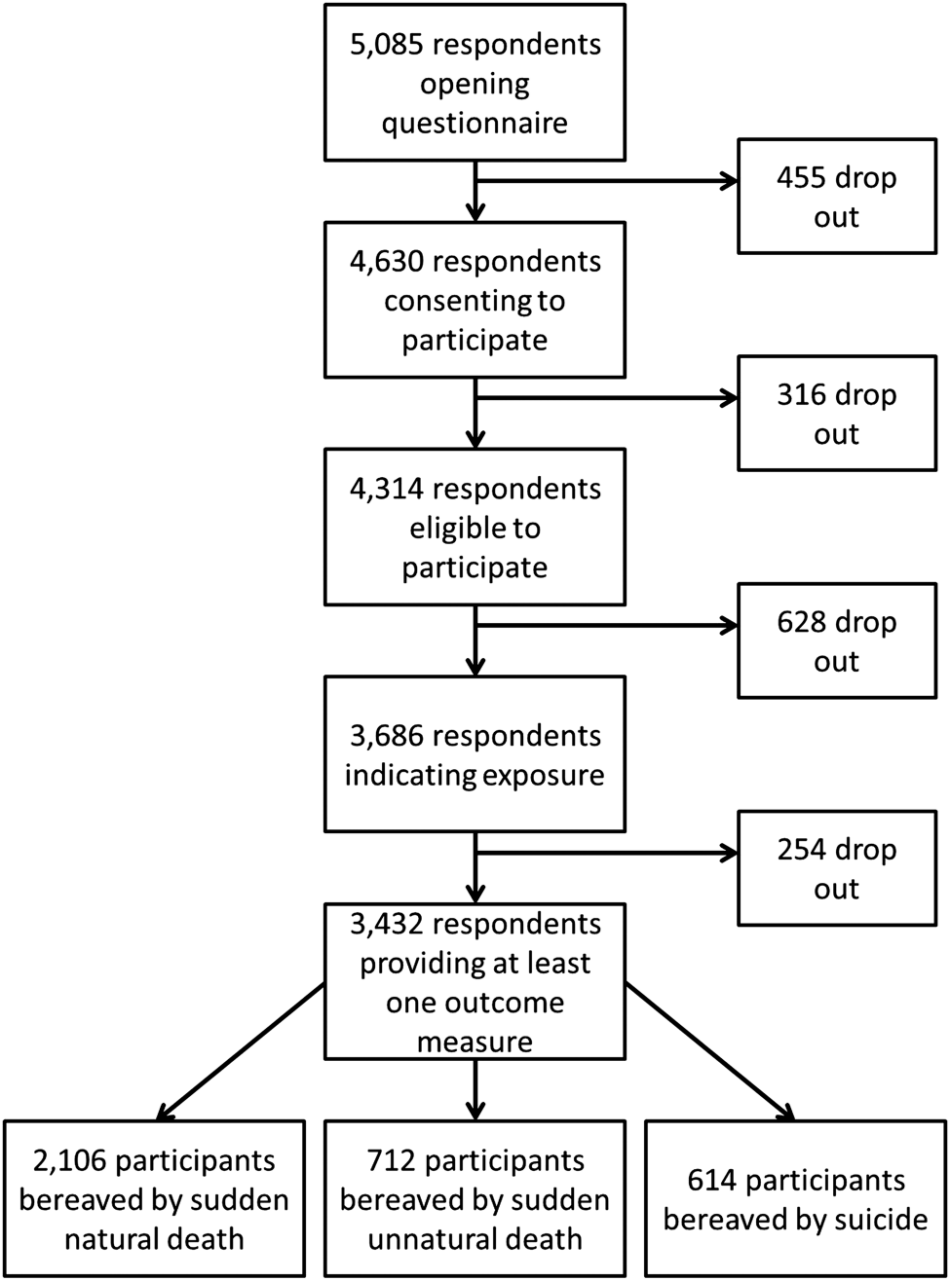
Participant flow.

**Figure 2:**
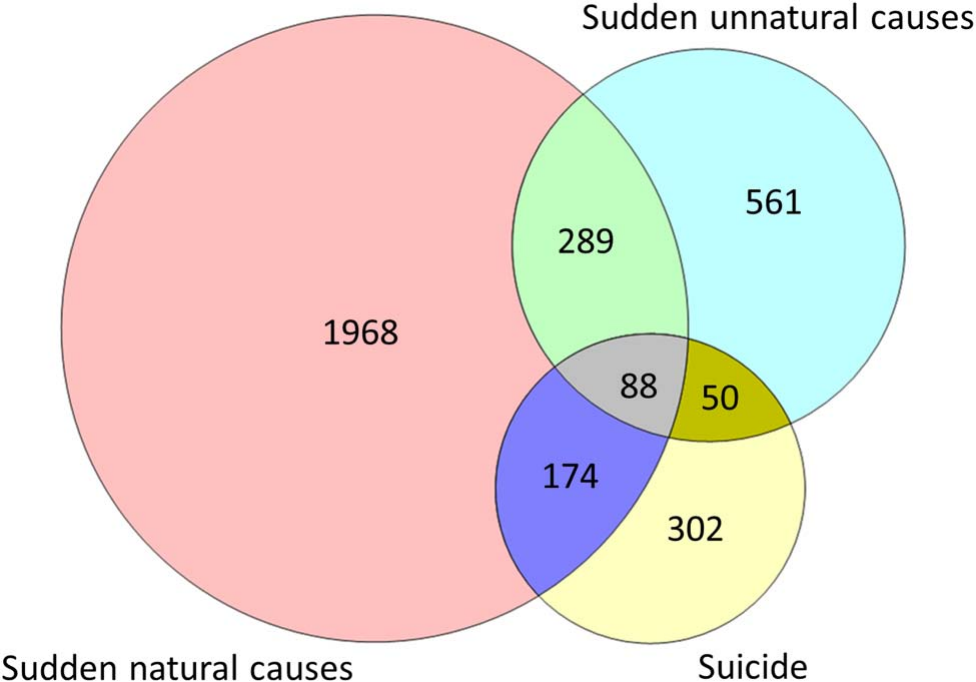
Euler diagram showing the combinations of exposures in eligible sample of 3432 respondents.

A total of 5085 people of the 659 572 sampled responded to the questionnaire by clicking on the survey link, with 91% consenting to participate, and 68% (n=3432) fulfilling eligibility (see figure 1). Overall 18% had been exposed to more than one mode of sudden bereavement (see figure 2), which was significantly more common in the group bereaved by suicide (see table 1). Clustering of participants within the 37 HEIs was minimal for primary outcomes (see table 1). Missing data for model covariates and outcomes were less than 7%.

**Table 1:** Characteristics of participants by type of bereavement exposure

Participants bereaved by	Sudden natural death (n=2106)	Sudden unnatural death (n=712)	Suicide (n=614)	Total (n=3432)	p Value*
*Sociodemographic characteristics*					
Gender,† n (%)					
Female	1709 (81)	576 (81)	499 (81)	2784 (81)	0.982
Missing	1 (<1)	0 (0)	0 (0)	1 (<1)	
Age of participant (years)† mean (SD)	24.9 (6.3)	25.2 (6.3)	25.2 (6.0)	25.0 (6.3)	0.069
Self-defined ethnicity, n (%)					
White	1877 (89)	645 (91)	562 (92)	3084 (90)	0.154
Non-white	228 (10)	65 (9)	52 (9)	345 (10)	
Missing	1 (<1)	2 (<1)	0 (0)	3 (<1)	
Socioeconomic status†,‡ n (%)					
Social classes 1.1 and 1.2	603 (29)	224 (32)	176 (29)	1003 (29)	0.179
Social class 2	684 (33)	234 (33)	204 (33)	1122 (33)	
Social class 3	259 (12)	77 (11)	68 (11)	404 (12)	
Social class 4	90 (4)	34 (5)	32 (5)	156 (5)	
Social classes 5, 6, 7 and 9	409 (19)	115 (16)	113 (18)	638 (19)	
Missing	61 (3)	27 (4)	21 (3)	109 (3)	
Educational status, n (%)					
No academic qualifications	2 (<1)	2 (<1)	0 (0)	4 (<1)	0.013
Attained maximum GCSE equivalent	33 (2)	8 (1)	12 (2)	53 (2)	
Attained maximum A level equivalent	929 (44)	276 (39)	243 (40)	1448 (42)	
Attained maximum degree equivalent	763 (36)	266 (37)	217 (35)	1246 (36)	
Attained postgraduate degree	373 (18)	158 (22)	142 (23)	673 (20)	
Missing	6 (<1)	2 (<1)	0 (0)	8 (<1)	
Student status, n (%)					
Student	1797 (85)	613 (86)	526 (86)	2936 (86)	0.822
Staff	253 (12)	78 (11)	68 (11)	399 (12)	
Both	55 (3)	21 (3)	20 (3)	96 (3)	
Missing	1 (<1)	0 (0)	0 (0)	1 (<1)	
Measure of current social support,§ n (%)					
No lack of perceived social support	1234 (59)	411 (58)	345 (56)	1990 (58)	0.740
Moderate lack of perceived social support	549 (26)	197 (28)	168 (27)	914 (27)	
Severe lack of perceived social support	323 (15)	102 (14)	100 (16)	525 (15)	
Missing	0 (0)	2 (<1)	1 (<1)	3 (<1)	
*Clinical characteristics*					
Family history of psychiatric problems, n (%)					
Yes	1243 (59)	434 (61)	412 (67)	2089 (61)	0.002
Missing	153 (7)	41 (6)	39 (6)	233 (7)	
Other family history of suicide,† n (%)					
Yes	123 (6)	41 (6)	53 (7)	217 (6)	0.038
Missing	158 (8)	43 (6)	40 (7)	241 (7)	
Prebereavement suicidal thoughts¶ n (%)					
Yes	584 (28)	178 (25)	185 (30)	947 (28)	0.076
Missing	148 (7)	39 (6)	40 (7)	227 (7)	
Prebereavement suicide attempt†,** n (%)					
Yes n (%)	125 (6)	28 (4)	49 (8)	202 (6)	0.007
Missing n (%)	154 (7)	40 (6)	40 (7)	234 (7)	
Prebereavement non-suicidal self-harm†,††					
Yes	400 (19)	121 (17)	141 (23)	662 (19)	0.016
Missing	154 (7)	40 (6)	40 (7)	234 (7)	
Postbereavement suicidal thoughts‡‡ n (%)					
Yes	911 (43)	322 (45)	299 (49)	1532 (45)	0.064
Missing	148 (7)	39 (6)	40 (7)	227 (7)	
Postbereavement suicide attempt§§ n (%)					
Yes	112 (5)	42 (6)	56 (9)	210 (6)	0.003
Missing	154 (7)	40 (6)	40 (7)	234 (7)	
Postbereavement non-suicidal self-harm, n (%)					
Yes	438 (20)	149 (21)	151 (25)	738 (22)	0.127
Missing	154 (7)	40 (6)	40 (7)	234 (7)	
Prebereavement depression† n (%)					
Yes	370 (18)	129 (18)	143 (23)	642 (19)	0.005
Missing	85 (4)	21 (3)	24 (4)	130 (4)	
Personality disorder screen positive¶¶ n (%)					
Yes	743 (35)	227 (32)	225 (37)	1195 (35)	0.082
Missing	131 (6)	31 (4)	33 (5)	195 (6)	
*Characteristics of the bereavement*					
Kinship to the deceased† n (%)					
Blood-related	1786 (85)	351 (49)	296 (48)	2433 (71)	<0.001
Non-blood-related	313 (15)	356 (50)	317 (52)	980 (29)	
Missing	7 (<1)	5 (1)	1 (<1)	13 (<1)	
Age of the deceased mean (SD)	55.1 (21.5)	31.0 (17.4)	31.9 (15.2)	45.9 (22.8)	<0.001
Years since bereavement† mean (SD)	4.8 (5.3)	5.3 (5.4)	5.1 (5.0)	5.0 (5.3)	0.140
Exposure to >1 mode of sudden bereavement, yes, n (%)	138 (7)	151 (21)	312 (51)	601 (18)	<0.001
Perceived stigma of the bereavement*** mean (SD)	11.9 (3.8)	12.3 (4.0)	14.0 (4.3)	12.3 (4.0)	<0.001

*p Values for group comparisons excluding missing values, using a two-sided significance threshold of p=0.05.

†Pre-specified confounding variable used in adjusted model.

‡Socioeconomic status using the five categories from UK Office for National Statistics.

§Measure of current social support from APMS.^19^

¶Values for each exposure group exceeded the maximum lifetime prevalence of suicidal ideation (20.6%) in any corresponding age group within the APMS 2007 household sample.^19^

**Values for each control group were less than the maximum lifetime prevalence of suicide attempt (7.3%) in any corresponding age group within the APMS 2007 household sample.^19^

††Values for each exposure group exceeded the maximum lifetime prevalence of non-suicidal self-harm (12.4%) in any corresponding age group within the APMS 2007 household sample.^19^

‡‡ICC=0.008 for suicidal thoughts (n=37 clusters/HEIs) indicating low within-cluster correlation of responses.

§§ICC=0.047 for suicide attempt (n=37 clusters/HEIs) indicating low within-cluster correlation of responses.

¶¶SAPAS-SR screen for personality disorder.^20^

***Stigmatisation subscale of the Grief Experience Questionnaire.^6^

APMS, Adult Psychiatric Morbidity Survey; ICC, intracluster correlation coefficient; SAPAS-SR, Standardised Assessment of Personality – Abbreviated Scale Self-Report.

### Participant characteristics

The sample was primarily female, white and blood-related to the deceased (see table 1). Of those reporting the loss of a non-blood-related contact, 74% described them as a friend, 11% a partner, 4% an ex-partner and 12% a step/adoptive/in-law family member. There were no statistically significant differences between the exposure groups in relation to mean age, gender, self-defined ethnicity, socioeconomic status, level of current social support or personality difficulties. People bereaved by suicide were significantly more likely to report prebereavement psychopathology, and a family history of psychiatric problems. Those bereaved by sudden unnatural causes or by suicide reported a lower mean age of the deceased than those bereaved by sudden natural causes, and were also less likely to report the loss of a blood relative. The mean time elapsed since bereavement was 4.9 years (SD=5.3; range=1 day to 30 years), with no evidence for group differences. In each exposure group, the prevalence of prebereavement suicidal thoughts and non-suicidal self-harm (but not suicide attempt) exceeded estimates for UK population norms in corresponding age groups.^19^

### Bereavement by suicide compared with that by sudden natural causes

In comparison with bereavement by sudden natural causes (see table 2), those bereaved by suicide had a greater probability of postbereavement suicide attempt (adjusted OR (AOR)=1.65; 95% CI 1.12 to 2.42; p=0.012), but not of suicidal ideation. The suicide-bereaved group also had a greater probability of occupational drop-out (AOR=1.80; 95% CI 1.20 to 2.71; p=0.005), but there was no evidence for group differences in postbereavement non-suicidal self-harm, depression or social functioning.

**Table 2:** Estimates of the relationship between postbereavement outcomes and bereavement exposure (sudden natural death as reference category)

Exposure group	Sudden natural death (n=2106)		Sudden unnatural death (n=712)					Suicide (n=614)				
	Prevalence n (%)	OR (reference)	Prevalence n (%)	Unadjusted OR* (95% CI)	p Value†	Adjusted‡ OR* (95% CI)	p Value†	Prevalence n (%)	Unadjusted OR* (95% CI)	p Value†	Adjusted‡ OR* (95% CI)	p Value†
Primary outcomes												
Postbereavement suicidal ideation	911 (43)	1	322 (45)	1.04 (0.87 to 1.25)	0.670	0.97 (0.80 to 1.18)	0.740	299 (49)	1.27 (1.05 to 1.54)	0.019	1.13 (0.92 to 1.39)	0.237
Postbereavement suicide attempt	112 (5)	1	42 (6)	1.09 (0.74 to 1.61)	0.656	1.11 (0.73 to 1.68)	0.621	56 (9)	1.76 (1.25 to 2.49)	0.001	1.65 (1.12 to 2.42)	0.012
Secondary outcomes												
Postbereavement non-suicidal self-harm	438 (20)	1	149 (21)	1.00 (0.81 to 1.25)	0.980	1.06 (0.82 to 1.37)	0.655	151 (25)	1.29 (1.04 to 1.61)	0.021	1.28 (0.98 to 1.66)	0.066
Postbereavement depression	647 (31)	1	249 (35)	1.20 (0.99 to 1.45)	0.059	1.22 (0.98 to 1.53)	0.071	180 (29)	0.94 (0.77 to 1.15)	0.553	1.03 (0.81 to 1.30)	0.840
Postbereavement occupational drop-out	96 (5)	1	44 (6)	1.41 (0.96 to 2.07)	0.079	1.56 (1.04 to 2.35)	0.033	48 (8)	1.66 (1.14 to 2.43)	0.009	1.80 (1.20 to 2.71)	0.005
Poor current social functioning	557 (27)	1	178 (25)	0.91 (0.74 to 1.11)	0.354	0.92 (0.73 to 1.15)	0.443	200 (33)	1.41 (1.15 to 1.73)	0.001	1.33 (1.06 to 1.67)	0.012

*Estimate obtained using *xtlogit* command in Stata.

†Two-sided significance threshold of p=0.05 for primary outcomes, and p=0.01 for secondary outcomes.

‡Adjusted for age, gender, socioeconomic status, prebereavement depression, prebereavement suicide attempt, prebereavement non-suicidal self-harm, other family history of suicide (excluding index bereavement), years since bereavement and kinship to the deceased. For each model, exposure group sizes exceeded the 466 respondents required for adequate power, even when using complete case analysis.

Comparison between bereavement by sudden unnatural causes and the reference category of adults bereaved by sudden natural causes showed no evidence for any group differences.

### Bereavement by suicide compared with that by sudden unnatural causes

When directly compared with bereavement by sudden unnatural death, adults bereaved by suicide had a similar probability of postbereavement suicidal ideation and suicide attempt (see table 3). The probability of poor social functioning was significantly greater in adults bereaved by suicide (AOR=1.46; 95% CI 1.12 to 1.89; p=0.005), but there were no differences in postbereavement non-suicidal self-harm, depression or occupational drop-out.

**Table 3:** Estimates of the relationship between postbereavement outcomes and bereavement exposure (suicide vs sudden unnatural death)

Exposure group	Sudden unnatural death (n=712)		Suicide (n=614)				
	Prevalence n (%)	OR (reference)	Prevalence n (%)	Unadjusted OR* (95% CI)	p Value†	Adjusted‡ OR* (95% CI)	p Value†
Primary outcomes							
Postbereavement suicidal ideation	322 (45)	1	299 (49)	1.22 (0.97 to 1.53)	0.087	1.17 (0.93 to 1.48)	0.189
Postbereavement suicide attempt	42 (6)	1	56 (9)	1.62 (1.04 to 2.50)	0.032	1.48 (0.94 to 2.33)	0.089
Secondary outcomes							
Postbereavement non-suicidal self-harm	149 (21)	1	151 (25)	1.29 (0.99 to 1.68)	0.061	1.21 (0.89 to 1.63)	0.222
Postbereavement depression	249 (35)	1	180 (29)	0.78 (0.61 to 1.00)	0.049	0.84 (0.64 to 1.10)	0.197
Postbereavement occupational drop-out	44 (6)	1	48 (8)	1.18 (0.75 to 1.84)	0.473	1.15 (0.73 to 1.82)	0.541
Poor current social functioning	178 (25)	1	200 (33)	1.56 (1.21 to 2.00)	0.001	1.46 (1.12 to 1.89)	0.005

*Estimate obtained using xtlogit command in Stata.

†Two-sided significance threshold of p=0.05 for primary outcomes, and p=0.01 for secondary outcomes.

‡Adjusted for age, gender, socioeconomic status, prebereavement depression, prebereavement suicide attempt, prebereavement non-suicidal self-harm, other family history of suicide (excluding index bereavement), years since bereavement and kinship to the deceased. For each model, exposure group sizes exceeded the 466 respondents required for adequate power, even when using complete case analysis.

### Kinship as a potential effect modifier

Tests for an interaction between bereavement exposure and kinship to the deceased found that none of the significant or non-significant associations between suicide bereavement and adverse outcomes were modified by relatedness. This was the case even when excluding the 253 respondents who reported the death of a partner, ex-partner or non-blood relative, to describe associations in a group bereaved by peer death.

### Stigma as a potential confounder

Adding stigma scores to adjusted models for significant associations between suicide bereavement and adverse outcomes attenuated ORs, as predicted, with no evidence for group differences between those bereaved by suicide and those bereaved by sudden natural causes in postbereavement suicide attempt (AOR=1.11; 95% CI 0.74 to 1.67; p=0.610) or occupational drop-out (AOR=1.36; 95% CI 0.89 to 2.09; p=0.156), or between those bereaved by suicide and those bereaved by sudden unnatural causes in terms of poor social functioning (AOR=1.06; 95% CI 0.80 to 1.41; p=0.667).

### Sensitivity analyses

Main findings were unchanged after sensitivity analyses simulating worst-case and best-case scenarios for missing values, and after those simulating other potential biases, suggesting that any biases introduced had not resulted in an underestimation or overestimation of the risks.

#### Post hoc sensitivity analyses

The magnitude and direction of the association between suicide bereavement and suicide attempt (compared with bereavement by sudden natural causes) were similar after excluding 769 participants bereaved by the death of someone aged over 60 (AOR=1.78; 95% CI 1.16 to 2.71; p=0.008), to exclude deaths that might be less unexpected. They were also unchanged when excluding 399 staff (AOR=1.73; 95% CI 1.16 to 2.59; p=0.007), and in a women-only sample (AOR=1.66; 95% CI 1.09 to 2.53; p=0.018). When compared with women bereaved by sudden unnatural causes, women bereaved by suicide had an increased probability of postbereavement suicide attempt (AOR=1.71; 95% CI 1.04 to 2.85; p=0.036), whereas in the full sample no association was found.

When taking into account the higher prevalence of repeated exposure to sudden bereavement in the suicide bereaved group, ORs were attenuated and no significant findings remained. There was therefore no evidence of group differences between the suicide bereaved group and those bereaved by sudden natural causes in relation to postbereavement suicide attempt (AOR=1.53; 95% CI 0.99 to 2.35; p=0.054) or occupational drop-out (AOR=1.54; 95% CI 0.98 to 2.43; p=0.062), or between those bereaved by suicide and those bereaved by sudden unnatural causes in relation to poor social functioning (AOR=1.41; 95% CI 1.07 to 1.84; p=0.013).

## Discussion

Our main finding was of a specific association between bereavement by suicide and subsequent suicide attempt among young adults who experience sudden bereavement. This was not attributable to prebereavement suicidality, despite higher rates of prebereavement psychopathology; a finding in keeping with the literature^1^ and suggestive of shared familial and environmental risk. Previous studies using non-bereaved controls or heterogeneous bereaved controls were not able to rule out the possibility that exposure to *any* sudden bereavement explains adverse outcomes. Our study supports a specific association between suicide bereavement and suicide-related outcomes, justifying the inclusion of people bereaved by suicide in national suicide prevention strategies. This study also provides the first evidence that blood relatedness to the deceased does not modify the association between suicide bereavement and suicide attempt, confirming that risk also applies to adults bereaved by peer suicide. Such findings must be interpreted in the context of a highly educated sample, in which exposure to violent losses may be lower than in a more nationally representative (but harder to recruit) sample.

The absence of an association between suicide bereavement and suicidal ideation or depression is striking, as is the high prevalence of prebereavement and postbereavement suicidal ideation and depression in all three exposure groups. This may be explained by high baseline rates of depressive and suicidal thoughts among students,^26^ reducing the chances of detecting a difference. It is also possible that while suicidal thinking after sudden loss is common, suicide bereavement is particularly powerful in precipitating suicide attempt in a suicidal person, whether due to enhanced awareness of means, reduced fear of death or social modelling.^27^ The non-significant differences in the probability of suicidality and depression when comparing adults bereaved by suicide and by sudden unnatural causes are noteworthy, requiring further studies comparing outcomes in those bereaved by suicide and other unnatural causes.

The clinical implications of these findings are that clinicians assessing suicide risk should inquire not only about a history of suicide in blood relatives, but also in friends and non-blood relatives. Employers should be aware of the impact of suicide bereavement on occupational functioning, and make adjustments to promote workplace mental health. The associations between suicide bereavement and adverse outcomes became non-significant when adding perceived stigma. This is an indicator that stigma might be a marker for motivational moderators of suicidality after a negative life event, such as reluctance to seek help, thwarted belongingness or perceived burdensomeness.^27^ However, further investigation is warranted to determine whether stigma can be said to lie on the causal pathway. This study suggests a role for psychosocial interventions delivered after a potentially traumatic loss to address problem solving and help seeking, and the quality of community support. Although not prehypothesised, the associations were also attenuated by repeated exposure to sudden losses among the suicide bereaved. This is suggestive of a substantial contribution of familial and environmental risk factors for premature death shared with social networks, and a reduced fear of death due to habituation. This acquired capability to attempt suicide^27^ would require sensitive exploration in a clinical interview.

Our study is of policy importance in specifying that friends as well as relatives warrant support after a suicide, addressing the vagueness of suicide prevention strategies on how extensively to offer support.^1^ The WHO estimates that 800 000 people die by suicide annually,^28^ and 60 people are now understood to affected by each suicide death.^29^ This means that 48 million people are bereaved by suicide worldwide every year. Further research describing moderators of risk will help determine whether there is a rationale for screening members of this heterogeneous group. Trials are also needed to identify evidence-based interventions delivered after suicide bereavement to reduce the risk of suicide-related outcomes, including those that address stigma.

This study's key strengths are its national population-based sample size, and ability to access those who do not normally participate in research. It is the largest scale survey conducted in any country comparing self-reported suicide-related outcomes in those bereaved by suicide and other mortality causes. Previous studies using national registries have achieved larger sample sizes, but under-recorded exposures and lacked self-reported outcomes such as untreated suicide attempts.^10–12^ ^30^ ^30^ ^31^ Unlike previous surveys, we tested clear a priori hypotheses, accounted for prebereavement psychopathology, and used standardised measures for seven of eight outcomes. A precise sampling frame accessed a large community sample of young adults, otherwise under-represented in health research, while minimising the biases inherent to using help-seeking groups. Coroner misclassification of suicides as accidental deaths was less of a problem than in other studies as we used the respondent's perception of cause of death, with minimal potential for respondent misclassification. Levels of missing data within models were low, and results were robust to sensitivity analysis simulating non-response and possible selection biases. Chance findings were unlikely as group sizes exceeded the minimum required for adequate power and the significance threshold was more stringent for secondary outcomes than primary.

Lack of information on response might be a considered a limitation, but no method permitted accurate estimation of the bereaved denominator. It is reasonable to assume that most non-responders had not been exposed to sudden bereavement, and that a minority were ineligible by age. Our hierarchical approach to classifying suicide exposure may have overestimated the effect of suicide bereavement due to clustering of violent bereavements, but we did not measure number of exposures to each type of bereavement. Our definition of non-suicidal self-harm followed that used for establishing UK population norms,^19^ but may differ from others given wide international definitional variations. Recall bias may have influenced judgements about the onset and severity of difficulties, particularly among those bereaved by violent causes, with the potential to overestimate risks in these groups. Residual confounding is possible in relation to unmeasured variables such as financial hardship, social modelling, substance misuse and complicated grief (which was not measured as data collection preceded Diagnostic and Statistical Manual of Mental Disorders, 5th Edition (DSM-5)^32^). Non-response bias (from men, those most distressed), survivor bias and selection bias (favouring higher social classes) may have resulted in an underestimation of risks due to the higher probability of suicide-related outcomes in disadvantaged men^16^ and those worst affected by the loss. An HEI sample is not representative of all UK-based young adults, despite inclusion of diverse institutions, and this limits generalisability of findings to those not entering higher education. While selection bias and male non-response bias were equally distributed between the three exposure groups, the results of this study may be more generalisable to young bereaved women than men, and to the more highly educated. Nevertheless, the findings do constitute the best available evidence describing the impact of peer suicide on young adults using appropriate controls.

## Conclusions

Bereavement by suicide is a specific risk factor for suicide attempt when compared with bereavement due to sudden natural causes, whether blood-related to the deceased or not. As the association between suicide bereavement and suicide attempt is attenuated when taking into account perceived stigma, further investigation of the role of stigma and reduced help seeking is warranted. Such work will inform the development of acceptable interventions delivered after potentially traumatic losses. Our findings suggest that suicide risk assessment should extend screening for a family history of suicide to any history of suicide in non-blood relatives and friends. However, until we have evidence-based interventions for this group, the best ways of mitigating this risk of suicide attempt are unclear.
